# Exploring the role and function of trial steering committees: results of an expert panel meeting

**DOI:** 10.1186/s13063-015-1125-z

**Published:** 2015-12-30

**Authors:** Nicola L. Harman, Elizabeth J. Conroy, Steff C. Lewis, Gordon Murray, John Norrie, Matt R. Sydes, J. Athene Lane, Douglas G. Altman, Colin Baigent, Judith M. Bliss, Marion K. Campbell, Diana Elbourne, Stephen Evans, Peter Sandercock, Carrol Gamble

**Affiliations:** Medicines for Children Clinical Trials Unit, University of Liverpool, Liverpool, L12 2AP, UK; Department of Biostatistics, University of Liverpool, Liverpool, L69 3GA, UK; Centre for Population Health Sciences, Edinburgh University, Edinburgh, UK; Centre for Healthcare Randomised Trials (CHaRT), Aberdeen, UK; MRC Clinical Trials Unit at UCL, London, UK; London Hub for Trials Methodology Research, London, UK; Bristol Randomised Trials Collaboration Trials Unit, Bristol, UK; Centre for Statistics in Medicine, Nuffield Department of Orthopaedics, Rheumatology & Musculoskeletal Sciences, University of Oxford, Oxford, UK; Clinical Trial Service Unit & Epidemiological Studies Unit (CTSU), Nuffield Department of Population Health, University of Oxford, Oxford, UK; ICR-CTSU, Division of Clinical Studies, The Institute of Cancer Research, London, UK; Health Services Research Unit, University of Aberdeen, Aberdeen, UK; Department of Medical Statistics, London School of Hygiene and Tropical Medicine, London, UK; Faculty of Epidemiology and Population Health, London School of Hygiene and Tropical Medicine, London, UK; School for Clinical Sciences, University of Edinburgh, Edinburgh, UK

**Keywords:** Trials, oversight committee, trial steering committee, executive committee, clinical trial, data monitoring committee, terms of reference, randomised controlled trial

## Abstract

**Background:**

The independent oversight of clinical trials, which is recommended by the Medical Research Council (MRC) Guidelines for Good Clinical Practice, is typically provided by an independent advisory Data Monitoring Committee (DMC) and an independent executive committee, to whom the DMC makes recommendations. The detailed roles and function of this executive committee, known as the Trial Steering Committee (TSC), have not previously been studied or reviewed since those originally proposed by the MRC in 1998.

**Methods:**

An expert panel (n = 7) was convened comprising statisticians, clinicians and trial methodologists with prior TSC experience. Twelve questions about the role and responsibilities of the TSC were discussed by the panel at two full-day meetings. Each meeting was transcribed in full and the discussions were summarised.

**Results:**

The expert panel reached agreement on the role of the TSC, to which it was accountable, the membership, the definition of independence, and the experience and training needed. The management of ethical issues, difficult/complex situations and issues the TSC should not ask the DMC to make recommendations on were more difficult to discuss without specific examples, but support existed for further work to help share issues and to provide appropriate training for TSC members. Additional topics discussed, which had not been identified by previous work relating to the DMCs but were pertinent to the role of the TSC, included the following: review of data sharing requests, indemnity, lifespan of the TSC, general TSC administration, and the roles of both the Funder and the Sponsor.

**Conclusions:**

This paper presents recommendations that will contribute to the revision and update of the MRC TSC terms of reference. Uncertainty remains in some areas due to the absence of real-life examples; future guidance on these issues would benefit from a repository of case studies. Notably, the role of a patient and public involvement (PPI) contributor was not discussed, and further work is warranted to explore the role of a PPI contributor in independent trial oversight.

**Electronic supplementary material:**

The online version of this article (doi:10.1186/s13063-015-1125-z) contains supplementary material, which is available to authorized users.

## Background

Independent oversight committees are recommended in the management of clinical trials. The Medical Research Council (MRC) Guidelines for Good Clinical Practice (1998) defines a three-committee oversight structure that has been widely adopted in the UK: the Trial Management Group (TMG), the Data Monitoring Committee (DMC) and the executive Trial Steering Committee [1]. The TMG is responsible for the day-to-day delivery and conduct of the trial; the DMC role is to review safety and efficacy data and make recommendations to an executive group; and the TSC is the executive decision-making group that considers the recommendations from the DMC. Members of the TMG are not independent from the trial; all members of the DMC are independent; and the TSC has both independent members and members who are also part of the TMG.

Extensive work as part of the DAMOCLES study [2] has been done to establish why and when DMCs are needed; their roles, responsibilities and structure; their organisation; and their decision-making and reporting arrangements. The DAMOCLES study concluded that the role of the DMC, as indicated within their Charter, is not to make decisions about the trial but rather to make recommendations to an appropriate executive committee [3]. The appropriate executive committee that considers these recommendations is the Trial Steering Committee (TSC), yet, to date, no such in-depth project establishing the role, responsibilities, and organisation of this committee has been conducted. The remit, objectives and functionality of the TSC is usually described and documented in the TSC terms of reference. The MRC TSC terms of reference, an appendix of the 1998 MRC guidelines for Good Clinical Practice [1], describe the role of the TSC as providing the overall supervision of the trial. These guidelines have been used in the development of local terms of reference by the majority of UK Clinical Trials Units (CTUs) who, in a recent survey [4], reported having TSC terms of reference in place. However, many modified the MRC guidelines from other sources and their own experiences, suggesting that the TSC terms of reference should be updated.

We aimed to explore the roles and responsibilities of the TSC from the perspective and experience of an expert panel of clinicians, trial methodologists and statisticians, and to use this to inform revision of the MRC TSC terms of reference. This was one of three projects funded by the MRC Hubs for Trials Methodology Research Network on Trial Steering Committees. One of these funded projects focused on diagnostic studies, whilst this project and a separate ethnographic study focused on TSCs for clinical trials. A member of the ethnographic study (JAL) acted as an intermediary between the ethnographic project and the current study.

## Methods

The study management group (NH, EC, SL, JN, GM, MS, JAL, and CG) submitted nominations for expert panel membership with subsequent voting. Individuals with two or more votes (n = 10) were invited to join the panel, seven accepted (DGA, CB, JB, MKC, DE, SE, and PS) and attended one (n = 4) or both (n = 3) of the expert panel meetings. Members of the study management group also attended and participated in each meeting. Panel expertise included statisticians, trial methodologists, clinicians, contributors to the DAMOCLES study and CTU Directors/Deputy Directors. Members of the expert panel and attending study team had previously acted as members (independent and non-independent) of trial oversight committees.

Prior to the first meeting, a list of questions relating to the role and responsibilities of the TSC was developed. There were twelve categories of questions, some of which included additional sub-questions (Table 1). They were based on the questions used by the DAMOCLES study [5] and included additional questions developed by the study team using their personal experience and responses to the CTU survey [4]. These additional questions related to interactions between the TSC and the DMC. In particular, they addressed whether there are issues that the TSC should not ask the DMC to comment on and also what the role of the TSC is in situations where there is no DMC. The increasing call for data sharing, for which the MRC also has a policy [6], led to the inclusion of a question on the role of the TSC in the response to requests for sharing of data.

**Table 1:** Key questions about the role of the Trial Steering Committee (TSC). Topics prioritised for discussion at the meeting are marked with an asterisk

1)* What is the role/remit of the TSC?
2) The membership of the TSC: a. What should the membership of the TSC be with respect to size, members, and the choice of the Chair? b. Do TSCs always need independent members and, if so, how many? c. What is the definition of independence? d. Should Funders be able to reject TSC members? e. What, if any, are the implications of the same statistician being involved in production of DMC report (knowledge of unblinded comparative data) and being on the TSC?
3) What sort of experience and training should TSC members have?
4) What is the role of the TSC prior to trial recruitment?
5)* What material should be available to a TSC (meeting report contents)? a. Should a TSC see any data split by treatment groups? b. Should external evidence be incorporated and how? c. How does the material differ for TSCs that cover multiple trials?
6) What are the questions that the TSC should not ask the DMC to make recommendations on, for example, change to primary outcome or target sample size (other than pre-planned internal pilot)?
7) If there is no DMC, what is impact on the role/remit of the TSC?
8) How should decisions be reached within the TSC?
9)* How should ethical issues be handled in TSCs? For example, what if higher rates of adverse events were observed in one site or with one surgeon or an occurrence of a potential serious breach has occurred, for example, in dosing?
10)* What should be done in difficult/complex situations? a. What should be done when there is a recommendation from the DMC to stop the trial, who should be unblended, and what are the implications if the trial then does not stop? b. What should be done when decisions are made on site closures guided by performance? c. What should be done when Funders stop/withdraw funding? d. What should be done when an external body wants access to the data while the trial is on-going, for reasons such as design of a new trial or interest from industry?
11) What should the TSC’s role be concerning publications? Please consider publications prior to the main trial report and subsequent articles.
12) What should the TSC’s role be concerning requests for data sharing? Please consider requests prior to and following the main publications.

Questions were circulated to the expert panel, and members were asked to independently identify their five priority questions for discussion at the upcoming face-to-face meeting. Four questions were prioritised by at least 70 % of the group (Table 1). Members of the expert panel were not invited to add additional questions prior to the meetings but rather were encouraged to discuss the questions and to discuss broader issues should they feel that there were important uncertainties to address.

Following the first meeting, all agreed that a follow-up meeting to discuss the remaining questions was warranted and would allow all questions to be considered.

Before the meeting, a summary of relevant sections from guidance identified through the CTU survey, a literature search, and requests made to national and international funding bodies was provided. Specifically, these documents were guidance from the DAMOCLES report [2], MRC Guidelines for Good Clinical Practice [1], and the TSC requirements from the National Institute of Health Research (NIHR) Health Technology Assessment (HTA) and Efficacy and Mechanism Evaluation (EME) funding streams [7] (Additional file 1).

Each meeting was recorded and transcribed in full. Following the meeting, NH and CG reviewed the transcripts and categorised the discussions based on the questions in Table 1; separate categories were generated where other relevant topics were discussed. Discussions were then summarised and fed back to the expert panel for comment and agreement. Fully anonymised direct quotes are presented.

## Results

All twelve categories of questions were discussed by the expert group. Summary results for each question are described.

### What is the role/remit of the TSC? (Question 1)

The role of the TSC includes making decisions, endorsing TMG actions, and providing advice. The TSC may make recommendations to the Funder and/or Sponsor of a trial as well as to the trial team.

‘ …they may make decisions, they may make endorsements and they may provide advice, so there are three different things that they would consider doing and they consider the interests in making those conclusions, the interests of the trial, the Funder and the Sponsor, so that they’re looking at it as a complete package rather than being responsible to any one of those three things’.

All acknowledged that there were different TSC models in place both in the UK and internationally and that one size does not necessarily fit all clinical trials. JAL presented the preliminary results of an ethnographic study [8] in which the role of the independent TSC members had been described as a ‘critical friend’. The expert panel agreed with this idea, identifying independent TSC members as experienced, knowledgeable individuals who ask the bigger questions that the investigators being so close to the trial may not think through. The role was described as not only to ask these bigger questions but also to provide advice and support to facilitate troubleshooting.

The hierarchy of the oversight committees was discussed. There was agreement that the TSC, being responsible for decision-making, is the highest level oversight committee. In contrast to existing guidance, which described the TSC as supervisory, members of the expert panel agreed that the term ‘oversight’ was more appropriate.

There was much debate on the role of the TSC, its responsibilities, and who the committee ‘worked’ for.

‘…there is that balance of who are they working for, to protect, and to what aim? … one is to … make sure,…, that the Funder isn’t wasting its money; the other one is the Sponsor to make sure that legally the trial is being conducted to a good standard, but, ultimately, it’s to make sure that the trial can deliver high quality, to answer the question, … to make sure that it’s got some validity’.

Members of the expert group felt that the TSC did not work for one particular stakeholder of the trial but rather that they considered the interests of the trial holistically from the perspective of past, future, and current trial participants and patients; trialists; the Funder; and the Sponsor. This was in contrast with the major public Funders [7], who felt that the independent TSC members worked on their behalf, and as such, the Funder should have the power to appoint and dismiss members. In cases where public Funders consider the TSC to work on their behalf, the expert panel recommended that this responsibility be clearly specified in the terms of reference and invitations issued to independent members to join the TSC.

‘…in any terms of references there will have to be an explicit statement in there if it’s an HTA [Health Technology Assessment] trial that actually the TSC is working on behalf of the Funder if that’s the case… TSC members will have to sign up to that constraint…’

The panel felt that in commercial studies, it would make uncomfortable reading if the terms of reference stated that the TSC worked for the commercial funder. It is reasonable for the Funder’s interests to be represented, but the means of achieving this needs consideration. Perhaps, so as not to sway the balance of interests represented, not all independent TSC members should work for the Funder. However, this approach assumes that that the Funder’s interests are different from those of other stakeholders. This issue needs clarification and for Funders to explicitly state their ‘interests’. For example, for a public funding body, interests may, in fact, be more than solely financial and be heavily weighted towards participant rights and well-being, scientific integrity, and contribution to the evidence base.

### The membership of the TSC: What should the membership of the TSC be with respect to size, members, and the choice of Chair? (Question 2a)

The MRC guidance recommends a minimum TSC membership of an independent Chair and at least two other independent members, one or two (non-independent) principal investigators (meaning the Chief Investigator) and, where possible, a PPI contributor. The HTA guidance is more detailed, expecting the TSC to comprise an independent Chair, one or more independent clinicians, one or more independent statisticians/epidemiologists/diagnosticians and at least one PPI contributor, with independent members being a 75 % majority [7] (Additional file 1).

The expert panel agreed that the membership should include an independent Chair and at least two other independent members. Whilst including an independent statistician is recommended, the panel noted that there is a current paucity of suitably experienced statisticians in the UK. However, the panel agreed that this should not be used as an argument against recommending best practice.

Discussions suggested that a trial methodologist may be a suitable alternative to a statistician, provided that the role is clearly specified. Where issues arise during a trial that require alternative expertise, additional TSC members should be co-opted to provide advice and opinion in their field.

‘Also there’s no reason why a [trial] steering committee has to just be one group who will deal with all eventualities. There’s no reason why you couldn’t come to a situation and say, we’re uncomfortable with this, we need an expert on what happens when you have X happening, let’s co-opt someone in’.

However, co-opting may have implications on the length of time to make a decision on DMC recommendations. This is particularly important in the example given by a statistician when the DMC is recommending stopping the trial.

‘… quite often you don’t particularly need statistical skills, although it’s very valuable, but where you absolutely need statistical skills is if … any complex things come up, like a decision comes up from [the] DMC saying, I think we need to close the trial. So for me statistical expertise is essential on the TSC’.

‘ …you’ve got a trial that’s running and the DMC have made a recommendation to stop on the grounds of safety, then you’ve got a TSC considering that recommendation who, say for example, doesn't have a statistician involved, so they’ve [the TSC] got to go and find that expert and then we’ve got to get them informed about the trial, bring them up to speed…,it’s the impact that that might have on how long the trial continues for after the DMC has made its recommendation’.

The choice of Chair is important. Whilst they should have TSC experience, they do not necessarily need to be a clinician. As with DMCs, the Chair is sometimes chosen by the Sponsor or the investigators running the trial, and sometimes by the TSC themselves. In either case, the process of choosing the Chair should be clear and detailed in the invitation to join the TSC.

‘…the TSC Chair should have the experience and knowledge to be able to delve into those things - performance of the trial, all the statistics that enable you to see whether the trial is succeeding, compliance or adherence, … and direct the committee on what is needed if there are problems with those data’.

‘[in relation to the TSC Chair] It’s about the skills of the person rather than this, that or the other discipline…so that we can move away from that kind of expectation that it should be a clinical, and we can say openly it could either be clinical or non-clinical’.

### Do TSCs always need independent members and if so how many? (Question 2b)

Guidance on the membership of the TSC focuses on the independent membership, yet TSCs also include non-independent members, who are generally members of the TMG. However, the balance of membership should favour independent members to ensure that when consensus cannot be achieved, and voting is required, decisions reached are not unduly influenced by the trial team’s investment in the trial.

Additional trial team members and Sponsor or Funder representatives may attend the TSC to provide information only, but they are not considered members of the TSC; therefore, when a vote is required to reach a decision, these members should not be included in voting (see also question 8: How should decisions be reached within the TSC?).

No difference should exist between the aims of the non-independent and independent TSC members. Their relationship should be viewed as collaborative, with each member having respect for what the others contribute. Independent members offer experience, expertise, and a fresh look at the trial. On the other hand, non-independent members have an important role in the provision of in-depth knowledge of the day-to-day running of the trial. Nevertheless, the TSC should only be considered quorate for decision-making when the number of independent members outweighs the number of voting non-independent members. This approach offers more flexibility than the current HTA guidelines, which require the TSC to have a 75 % majority of independent members and a 67 % attendance of all appointed members to be quorate for decision-making. However, these recommendations do not take into account the specialities that should be represented or the appropriate composition and number of members present for decision-making.

‘If a trial is going to be steered, then the people who are the helm should have a say in how it’s steered … because they [the trial management group] have the most investment in the trial and they may be more knowledgeable than people on the [trial] steering committee about that trial, and I think they do have a stake in it and I feel it’s wrong to exclude them from the decision-making process’.

### What is the definition of independence? (Question 2c)

Independence was described by the expert panel as ‘not perceived to be influenced by the trial investigators, the Funder or the Sponsor’. To ensure that independence is maintained, the TSC independent members should not be from the same institution as the Sponsor, co-Sponsor, co-applicant, or the clinical trials unit involved in the management of the trial. When there is potential for a perception of influence, the members should clarify that it does not prohibit them from making impartial decisions. The expert panel recommended that independent members ask themselves the question ‘how will this work if things go wrong?’

Some UK Funders will not accept an independent TSC member if they are from the same institution as one of the investigators or recruiting sites. This criterion is potentially too restrictive, and expert panel members reported incidences where this definition was difficult to enforce and agreed that this should be a desirable criterion rather than mandatory. Instead, they proposed that those responsible for appointing TSC members, often the Funders, should be reassured of the absence of any working relationship that would influence the decision-making of the independent TSC member.

‘I've seen somewhere there's a recognition that somebody is from the same institution. It’s declared as an interest but it’s not seen as a problem so long as it’s upfront that that’s an issue. Especially if it’s a clinical/non-clinical definition of difference’.

‘I would prefer an experienced statistician from the same institution to an inexperienced statistician seen as more independent’.

### Should funders be able to reject TSC members? (Question 2d)

A consideration of both the appropriateness and independence of proposed independent members is required and for UK publically funded trials, this role is often taken on by the Funder and may be a condition of funding. Concerns were expressed that if a Funder has the right to both appoint and dismiss members, then the independence of the members from the Funder is compromised.

‘If you are appointed by the Funder and they have the right to unappoint you, then you are not really independent of them…’

The expert panel recommended that the Sponsor, given their overall responsibility for the trial [9, 10], should also be ultimately responsible for appointing and dismissing TSC members, an approach taken for DMC members within the ICH GCP [11]. However, it is important for all stakeholders to be confident in the expertise and independence of the proposed members. The expert panel recommended reaching agreement between stakeholders with provision of clear reasons should a stakeholder wish to reject or dismiss members.

If concerns are raised about the conduct or participation of an independent member, then these concerns should be discussed in the Trial Management Group. The Chief Investigator, as Chair of the TMG, should then lead on discussions with the TSC member to give the opportunity to acknowledge difficulties and act accordingly. If the concerns are maintained, then the decision to dismiss ultimately lies with the Sponsor.

### What, if any, are the implications of the same statistician being involved in production of the DMC report (with knowledge of unblinded comparative data) and being a member of the TSC? (Question 2e)

In an ideal scenario, where statistical resources are not limited, the panel agreed that the statisticians involved in the preparation of a DMC report that contains unblinded or comparative data should not be a voting member of the TSC. The key issue discussed was whether the statistician felt that their knowledge of the data in the DMC report would compromise their contributions and decision-making. Many of the expert panel felt that this would not be the case, provided that the statistician was experienced and confident to refuse to answer questions that could lead to inferences about effectiveness or safety between treatment arms. The panel noted that the knowledge of the unblinded statistician reflected a specific data cut and, therefore, a point in time that may no longer reflect the current situation in the trial.

‘…the statistician…has access to unblinded data but the result of that unblinded data will probably be different tomorrow than it is yesterday, so they only ever hold probably out of date information. So actually that’s why most statisticians can go through a whole trial because they’re never dealing with a set piece of information, it’s only as good as the last analysis’.

The discussion did not result in a firm answer but rather that the pros and cons of each circumstance should be considered; however, the panel agreed that the statistician producing the DMC report could attend the TSC meetings as an observer.

‘…it seems that there are considerations for that [statistician involved in the DMC report attending the TSC meeting] but I don’t think that it should be frowned upon or a case made for it, you should just consider what the pros and cons are for the practice of the trial’.

### What sort of experience, training, or preparation should TSC members have? (Question 3)

A TSC should represent a mix of skills and include both statistical/methodological and clinical inputs. All members of the TSC should have a commitment to the trial and skills that will help them act within their role as a TSC member, including being actively involved in trials themselves. Training to ensure the future capacity of experienced members is essential. Prior to acting as an independent member, the member should exhibit previous trials experience and acting as a non-independent member or an as observer for part of a wider trial team should be required.

‘…..but always try and have a junior person come and shadow or just observe, because I can remember when I first went to one of those [a TSC meeting], I hadn’t a clue what I was supposed to do, and I actually don’t think anyone else had much of a clue what they were meant to do either, but we have moved on a long way. But it is vital, yes there are too few people with a lot of experience, we need to… continually build up capacity’.

Members acting in an independent capacity for the first time may benefit from an independent mentor who would not attend the TSC meetings but would be bound by confidentiality and be willing to discuss issues with the appointed member.

Further discussion took place specifically about the experience of the independent TSC Chairperson. The expert panel considered the experience of the Chair to be particularly important and that the Chair, as in the DAMOCLES recommendations for DMCs, should have previous experience of TSC meetings and be able to facilitate meetings and enable effective interaction within the group.

‘…you need a range of skills, you need a strong Chairman who is experienced …, and who has a good relationship with the trial investigators and is able to speak to them, often about [difficult] issues…’

### What is the role of the TSC prior to trial recruitment? (Question 4)

The TSC is usually established after notification of a funding award, with the first meeting of the TSC taking place between gaining ethical approval and the start of recruitment. Like the DMC, the main role of a TSC prior to trial recruitment is to agree on the way in which they will operate and to review the trial protocol. Scheduling a joint first meeting of the TSC and DMC can be helpful. As indicated in DAMOCLES, due to the timing of this meeting, this first meeting usually involves protocol acceptance rather than proposing protocol changes. The expert panel considered the following statement from DAMOCLES to apply equally to TSCs: ‘All potential DMC members should have sight of the protocol/outline before agreeing to join the committee. Before recruitment begins, the trial will have undergone review by the Funder/Sponsor (for example, peer review for public sector trials) and scrutiny by other trial committees and a research ethics committee. Therefore, if a potential DMC member has major reservations about the trial (for example, the protocol or the logistics), he/she should report these to the trial office and may decide not to accept the invitation to join’.

‘…there's been peer review, there's been Funders’ changes. … And I think we shouldn’t, as a TSC, then undo that, because in a sense you are going back to the Funder and saying, well, should you have funded this?’

Attention should be given to the timing of the next TSC meeting following protocol acceptance; ideally, this meeting will be planned based on time elapsed rather than on patient numbers recruited. This approach to the timing of the meeting ensures that issues with trial opening, site opening, or recruitment can be addressed. The TSC should also consider themselves to be on standby throughout the trial should important issues arise, particularly if there is a planned DMC meeting.

‘I guess maybe you base it on time rather than on those first few patients coming in, because there may be something going on that means that recruitment is very slow, and that you want the TSC to be involved with’.

All agreed that review and approval of the trial protocol was essential and that the terms of reference should include a statement to acknowledge that members have read and agreed on trial conduct in accordance with a specified version of the trial protocol. Formal sign off of the protocol by the TSC, on the other hand, is not required.

### What material should be available to a TSC (meeting report contents)? Should a TSC see any data split by treatment groups? (Question 5a)

The expert panel debated the roles of the TSC and the DMC because the potential existed for the line between these different roles to be blurred with resulting duplication of effort. Specific reference was given to the checks of randomisation and review of baseline data split by treatment group; in this instance, the panel agreed that whilst the TSC would need to be assured that such checks had taken place, the actual completion of the checks was normally the responsibility of the DMC.

‘But you normally have… [DMC] dummy reports… within which there’s one section which is based on randomisation and the checks or issues with randomisation, so you know that the DMC have been supplied with the information …. So, for me, I think it sits with the DMC, and the DMC can then make a recommendation to the TSC who doesn't need to see that split by treatment group’.

All accepted that the TSC should not view post-baseline data split by treatment group, but an exception could be made for baseline data, provided that the justification was clear and that this was agreed on in advance by both the DMC and the TSC.

The panel also recommended that the TSC members are given the opportunity to comment on the proposed content of the routine DMC reports, prepared at agreed-upon stages of the trial by the trial statistician and provided to the DMC, so that the TSC members can be assured that the DMC is provided with all necessary information to check areas of concern. Yet, changes required by the DMC in response to viewing reports should not be fed back to the TSC in case this alerts the TSC to areas of concern that should remain confidential to the DMC.

During the trial, the TSC should be provided with a report that is based on the information included in the open section of the DMC report. Care should be taken to avoid unblinding of any party (TSC or TMG) by inclusion of data based on the timing of visits or safety profiles.

The first meeting of the TSC should agree on the content of TSC reports. The panel reviewed the example templates for TSC agendas and reports included in the MRC guidelines for Good Clinical Practice [1]. The content was agreed on with some minor additions, and a suggested template is provided in Table 2. A summary of core documents that the TSC should have opportunity to comment on, together with reference documents that should be made available to the TSC, are given in Table 3.

**Table 2:** Suggested template for Trial Steering Committee (TSC) meeting agendas and reports

1.	Title of trial
2.	Funding source(s) and Grant No.
3.	Sample size sought
4.	Protocol amendments and sub-studies
5.	Summary of the data monitoring committee (DMC) recommendations
6.	Date recruitment started
7.	Proposed date for recruitment end
8.	Actual recruitment rate versus target rate (by month/quarter)
9.	Acceptance rate as a proportion of the following:
	i) those invited to participate, and
	ii) if known, all eligible participants
10.	Percentage of participants proceeding through each trial stage to allow monitoring of the recruitment and retention, including missing outcome data. Not split by treatment/intervention arms.
11.	Quarterly/monthly forecasts of recruitment for the planned remainder of the trial
12.	Losses to follow-up as follows:
	i) as a proportion of those entered, and
	ii) per month/quarter
13.	Data management metrics: rate of returns, volume of queries, time to return, enhanced metrics via electronic data capture
14.	Number for whom follow-up has been completed successfully (or still being successfully followed-up)
15.	Overall withdrawal rate and level of withdrawal summarising those patients who have withdrawn from treatment but are still in follow-up and those who withdraw with no future contact.
16.	Summary of adherence to treatment/intervention. Not split by treatment/intervention arms.
17.	Summary of adverse events including type, for example, adverse events, serious adverse events and suspected unexpected serious adverse reactions
18.	Completeness of data collected
19.	Any available results (pooled)
20.	Summary of protocol deviations overall and by site
21.	Any organisational problems or other trial issues
22.	Issues specific to individual trials (to be specified by the Steering Committee)

**Table 3:** Summary of documentation that should be made available to the Trial Steering Committee (TSC) for approval or information

Document	Documents that require TSC approval	Documents that should be made to be available to the TSC but do not require approval	Timing of when document should be approved/made available
Trial Protocol	✓		Prior to trial opening and then available at all future trial meetings. Must be agreed on by TSC independent member as condition of TSC membership, but formal sign off of the protocol is not required.
Statistical Analysis Plan *(if an independent statistician is a member of the TSC then consider an independent blind review of the statistical analysis plan)*	✓		Approved prior to implementation and then made available on request at future meetings.
Sub-study proposals/protocols	✓		To be approved prior to submission for regulatory and REC approval if possible.
TSC terms of reference	✓		Prior to or at the first TSC meeting.
Protocol amendments –major/substantial amendment	✓		Prior to submission of the amendment to the Research Ethics Committee.
Protocol amendments –minor/non-substantial amendment		✓	Throughout the trial, as they arise or as part of the next scheduled meeting.
Trial publicity/promotion plan		✓	As a minimum, at the next TSC meeting following document approval by the TMG.
Data sharing agreement if in place		✓	As a minimum, at the next TSC meeting following document approval by the TMG.
Monitoring plan – to include information of the monitoring of data quality		✓	As a minimum, at the next TSC meeting following document approval by the TMG.
DMC report plan		✓	As a minimum, at the next TSC meeting following document approval by the TMG.
Open DMC report		✓	As a minimum, at the next TSC meeting following the DMC at which the report was presented or as agreed with the TSC if meeting frequency means that there will be as substantial delay in the TSC receiving this information.
Risk Assessment		✓	As a minimum, at the next TSC meeting following document approval by the TMG.
Publication policy		✓	As a minimum, at the next TSC meeting following document approval by the TMG.
Main trial publications		✓	Prior to submission
Participant information sheet and consent form *(important for TSC to review consent form and what participants are providing consent for)*		✓	As a minimum, at the next TSC meeting following document approval by the TMG.
CRFs/questionnaires/data collection tools		✓	As a minimum, at the next TSC meeting following document approval by the TMG.
DMC charter		✓	As a minimum, at the next TSC meeting following document approval by the DMC.

### What material should be available to a TSC (meeting report contents)? Should external evidence be incorporated and how? (Question 5b) How does this material differ for TSCs that cover multiple trials? (Question 5c)

Question 5b and 5c were not discussed in detail by the expert panel. With respect to external evidence, the panel agreed that it is the responsibility of the Chief Investigator (CI) and the trial team to provide information on new external evidence. There were limited examples of TSCs that cover multiple trials, and therefore, a case-by-case approach to the materials available should be taken in these circumstances, while using the suggested report content and documentation (Tables 2 and 3) as a starting point for discussions.

### What are the questions that the TSC should not ask the DMC to make recommendations on, for example, change to primary outcome or target sample size (other than pre-planned internal pilot)? (Question 6)

Discussion on question 6 was limited, and the expert panel noted that it was difficult to identify examples. The panel agreed that blinding of the TSC must be maintained, and that the TSC should not request the DMC to provide opinion on matters that could be influenced by their knowledge of comparative analyses. Examples of such matters include changes to primary outcome [12, 13], amendments to statistical analysis plans, and planned sample size.

### If there is no DMC, what is the impact on the role/remit of the TSC? (Question 7)

DAMOCLES describes circumstances where a DMC might not be required and states that this decision is made by the TSC, although input may also be needed from the Funder and Sponsor. The expert group noted that where there is no DMC, the TSC members should consider the justification and agree whether this is appropriate. Members of the TSC should only agree to membership if they are comfortable with the oversight structure in place for the trial.

Furthermore, the TSC should remain blinded in instances where there is no DMC, with only pooled data being reviewed. Where circumstances arise that cause the TSC concern, they should convene an emergency DMC. This emergency DMC should include independent members, who will review the un-blinded data and make a recommendation to the TSC, however the timeframe will need to be considered.

### How should decisions be reached within the TSC? (Question 8)

Wherever possible, the TSC should aim to reach consensus with all members (non-independent and independent) on the preferred plan of action for addressing an issue.

Voting may be necessary when consensus cannot be reached, but the voting rights of members should be made clear, and as already described, the number of independent members voting should outweigh the number of non-independent members. See question 2b.

### How should ethical issues be handled in TSCs? For example, what if higher rates of adverse events were observed in one site or with one surgeon or an occurrence of a potential serious breach, for example, dosing? (Question 9)

The TSC can have a role in contextualising complex ethical issues and identifying an appropriate course of action. The TSC also has a role in identifying early issues that might represent ethical concerns, such as deviation from the trial protocol, unexpected events that compromise patient safety, rights or wellbeing, and the potential impact of new external information. The panel recommends that when such issues are identified, they should be fed back to the trial team and a course of action agreed on in a case-by-case basis. For example, there may be some instances where further discussion takes place with the TSC, and a course of action is agreed on; alternatively, issues may need to be discussed with the trial Sponsor. If considered necessary by the TSC or the trial Sponsor, then issues may also be discussed further with the Ethics Committee that provided approval for the trial. An example was given where a trial participant had received an incorrect dose of the trial treatment, which constituted a breach of protocol. Ethical concerns were raised about whether or not to inform the patient. In this instance, the TSC were able to identify (taking into account the views of the PPI contributor) that the dose received by the participant was not above a standard daily dose, and that the potential worry that the patient might undergo, if informed, far outweighed any potential harms. As a result, agreement was reached by the TSC and the trial team that the patient did not need to be informed.

### What should be done when there is a recommendation from the DMC to stop the trial, who should be unblended, and what are the implications if the trial then does not stop? (Question 10a)

Here the expert panel reflected that in a situation when the DMC recommends stopping the trial for efficacy or safety, the TSC should ask the DMC whether their decision was unanimous and request that the independent TSC members be unblinded. Should the trial continue, the unblinded TSC members do not need to be replaced, but details of who has been unblinded should be included in the meeting minutes.

‘And do you think that that should happen? Because if people had a situation where the DMC said stop, and the independent members of the [Trial] Steering Committee said, ‘No, no, no, because you haven’t thought about the late benefit, look at this, let’s keep it going’, they’re all unblinded now, what do you do? Do you replace them even though they’re wise…?’

‘No, let them carry on.’

‘No, I think you just live with it.’

As noted previously, the TSC should be on standby at the time of a DMC meeting, so that if an issue arises, a meeting can be scheduled at short notice. The DMC Chair should communicate any urgent recommendations to either the Chief Investigator or TSC Chair who should then convene an urgent meeting to discuss the recommendations.

The DAMOCLES study [2] identified the need for planning how disagreements between the DMC and TSC should be managed, and this was also considered by the expert panel. All agreed that the process should include an escalation clause to describe how a difference of opinion on, for example, changes to the protocol or trial continuation should be managed. Options could include referral to the Sponsor, Funder or co-opted experts. The process should be clarified and agreed on by all members within the terms of reference.

### What should be done when decisions are made on site closures guided by performance? (Question 10b)

The TSC has a role in reviewing difficulties that arise in the course of the trial and providing feedback and guidance to the trial team on what corrective/preventative actions are necessary and how to implement them.

‘…the TSC, if they are made aware of problem sites, have a duty to do something about it’.

The TSC should consider patient safety and data integrity and be mindful of the relationships between the trial site and investigators when making recommendations of how to progress.

‘Yes. I mean it’s certainly not a key consideration but some trials are obviously run in very small communities and it can be really embarrassing for a Chief Investigator to close a centre’.

In such situations, the TSC can support the CI by relaying to the centre that the decision was that of the TSC.

### What should be done when Funders stop/withdraw funding? (Question 10c)

Although the Funder may withdraw funding from a trial, this does not necessarily mean that the trial should stop, as the decision to stop the trial ultimately lies with the Sponsor. In the event that funding is withdrawn, the TSC should consider whether they agree with trial closure, and if not, may offer advice and guidance to the trial team on continuation and alternative sources of funding.

### What should be done when an external body wants access to the data while the trial is on-going, for reasons such as the design of a new trial or interest from industry? (Question 10d)

The expert panel noted that the response to this question would depend on the specific request and the stage of the trial. As a result, specific guidance could not be generated, but instead, the panel suggested a general process whereby requests from Funders or external bodies for information should be sent to the Chair of the TSC. The TSC should then discuss the request and determine its appropriateness for the DMC.

‘Well surely it’s for the TSC to decide just… to consider requests for data and then to make judgements based on rational arguments,…it would be for each TSC to have a discussion about whether to provide data. And if the trial’s ongoing, …I think the assumption would be that you wouldn’t provide data, quite frankly, unless it’s really important…’

### What should the TSCs role be concerning publications? Please consider publications prior to the main trial report and subsequent articles. (Question 11)

Again, in line with DAMOCLES, [5] all agreed that being a named author on a trial publication does contravene the independence of the independent TSC members. However, the TSC should have the opportunity to comment on trial publications, and certainly, at a minimum, on the main trial report. The TSC membership, name and affiliation, should be included in the acknowledgements section of publications with the permission of the members.

The DAMOCLES study [5] also noted that some had suggested that the DMC should be responsible for ensuring that reporting occurs. The importance of ensuring timely reporting was echoed in the expert panel discussions who felt that the TSC had an ethical obligation to ensure that trial results were published. Examples were given where the TSC had helped to push publication by the trial team and also an example where the trial data was taken on by the DMC and subsequently reported with the Chair of the DMC as first author. All agreed these approaches were acceptable if, without them, the trial would remain unpublished. The feasibility of an oversight committee taking on the responsibility of publishing the results of the trial, if the team fail to do so, needs further consideration.

### What should the TSC’s role be concerning requests for data sharing? Please consider requests prior to and following the main publications. (Question 12)

The TSC has a role in reviewing requests for data sharing, particularly when requests, in line with the trial data sharing agreement, compete with the ongoing or planned work of the TMG. The TSC may not have a role in all requests for data sharing, only those that require careful consideration due to implications for ongoing data collection and planned analyses.

The expert panel remarked that the life span of the TSC should be taken into account when planning for future data requests. These are often submitted sometime after the trial has ended. A decision should be made as to whether the TSC would reconvene for such decisions or would be happy to pass this onto a central committee considering such requests across a portfolio of clinical trials, for example, within a clinical trials unit.

### Other uncertainties identified from discussions

Expert panel discussion identified further areas of uncertainty in the role and operation of the TSC. These are discussed below.

#### Lifespan of the TSC

The lifespan of the TSC was considered not only important for data-sharing considerations, as already discussed, but was also felt to need consideration when the TSC is in place for a feasibility trial that then progresses through to a pilot and/or main trial. In particular, they addressed whether the TSC is considered to maintain independence and continue to act as the TSC for the trial.

‘I think the example [NAME] has where somebody has been involved already, and was seen as independent at the start, I think that having a fixed rule that denotes you can’t continue is a mistake’

An example was also discussed on the role of the TSC when there is long-term follow-up post-intervention and the role of the DMC has concluded. All agreed that in these circumstances the oversight needs of the trial should be discussed and agreed on as part of the terms of reference.

#### Sponsorship

Legally, the Sponsor has the ultimate responsibility for a trial, and legal responsibility remains even when roles are delegated. However, discussions reflected uncertainty in the ability of some institutions to fulfil this role fully due to lack of expertise. In current practice, the Sponsor often delegates the decision of trial continuation to the TSC. All agreed that the Sponsor should formally document the responsibilities delegated to the TSC. Where decisions around continuation are not delegated to the TSC, the recommendations from the TSC should be submitted to the trial Sponsor.

#### Indemnity

The TSC is delegated responsibilities by the Sponsor, but it is often unclear whether the Sponsor also provides indemnity for the TSC members. The expert panel noted that professional indemnity for TSC membership may not be provided by their own academic or clinical institution, and that for public contributors or retired academics and clinicians, the situation was even less clear. No recommendations were made as to who should provide indemnity, but instead, the panel suggested that indemnity arrangements should be made clear in the TSC terms of reference.

#### Administration

More practical arrangements of the TSC were debated by the expert panel, and we have included recommended best practice in the administration of the TSC meetings. Where a TSC is convened for a single trial, meetings of the TSC should aim to be scheduled to take place 1 or 2 weeks after the DMC meeting. Where this is not possible, the Chair of both the TSC and the DMC should be informed, so that a joint decision on appropriate timing of meetings can be reached. The frequency of TSC meeting should be determined based on the trial risk assessment. An inexperienced TSC may prefer to meet more frequently, but this may have implications for the cost of the trial, including for resources, for organising the meetings, and for preparing the TSC reports. The expert panel suggested that the terms of reference include a recommended frequency of TSC meetings and the level of reporting required. Table 2 describes the content of the TSC reports and agendas. However, shorter, more focused reports may be appropriate for some meetings and would make best use of trial resources.

The Chair may choose to take the minutes of meetings, to delegate this to another independent member or to a member of the trial team, for example, the trial coordinator or trial administrator. Where the responsibility of writing minutes has been delegated, they should be reviewed by the Chair prior to circulation for approval by all present. The meeting agenda should be based on the TSC report; other items may be suggested by the trial team and agreed on with the TSC Chair. The timelines for sending documents or reports prior to the meeting should be agreed on in advance with the TSC members. The TSC may also convene as independent members only, particularly if there are sensitive issues to discuss, for example, the conduct of the TMG.

## Discussion

Some guidelines for the TSC terms of reference exist, but the panel felt that these are not exhaustive and may not consider some important issues in TSC conduct and membership. One of the key discussion points was the nature of independence and to whom the TSC was responsible; in particular, the panel addressed whether the latter influenced independence. A consistent and transparent approach to the appointment of the independent TSC members is needed. IFor many trials in the UK, the Funder is currently responsible for appointing and dismissing independent members, so input from these Funders will be crucial in defining for whom the TSC works, together with the criteria that should be applied when scrutinising suggested members. Interestingly some academic journals require the role of the Funder to be clearly specified in the trial manuscript, yet for many trials funded by the NIHR HTA programme, this is overlooked in funding statements [14–20].

We have identified areas where uncertainly remains and for which additional resources are required. Discourse for questions 10a-10c (what should be done in difficult or complex situations) was largely hypothetical due to the small number of examples available. This corresponds to the small number of papers identified in DAMOCLES as reporting what should be done in difficult situations in the context of the DMC [5]. The expert panel acknowledged that this gap could be addressed if there was a resource that allowed experiences to be shared. If members of TSCs and DMCs could submit examples, this would, over time, build a repository of issues and potential solutions relating to DMC and TSC roles independently and the interaction between the two committees. This could also help to identify additional examples of questions that the TSC should not ask the DMC to make recommendations on (question 6). The way in which the TSC and DMC interact, as described in the MRC guidelines, is well accepted, but this does not address situations where there is disagreement between the DMC and the TSC. Further work is needed to clarify the process to be followed and the wording of the escalation clause, which is included in the terms of reference, in these situations. The suggested repository may benefit from a widened scope to include anonymised examples of TSC/DMC disagreement and how they have been handled.

The current lack of training for independent members was highlighted. Requirements of independence mean that prior working relationships may exclude a number of potential independent members, thereby allowing few alternatives for experienced members if the field is small, as is the case for independent statisticians and/or trial methodologists. Capacity building will be essential to ensure a suitable pool of experts who have had the necessary training and experience to contribute to a TSC as an independent member; these experts must understand the workload, commitment and potential academic benefits of membership, for example, gaining knowledge from shared experience. Capacity building for statisticians will be addressed somewhat by an ongoing national initiative to create a database of statisticians (Search for Oversight Statisticians [21]). However, further thought on how best to build capacity for other disciplines is required. The model of TSC oversight on which recommendations have been made is that of a TSC acting for a single trial. However, results from the CTU survey [22] show that one quarter of UK Clinical Trials Units use at least one umbrella TSC, which has oversight of a number of trials that are taking place in the same clinical condition. Only one member of the expert panel had experience of such a model, so debate about the pros/cons and specific terms of reference consideration in these circumstances were limited. The resulting recommendation is that the content of the TSC reports and agendas should be considered on a case-by-case basis. However, further exploration of current practice with those CTU survey responders who use umbrella TSCs may help to distinguish specific considerations for this model of oversight.

Public representation on a TSC is recommended by both MRC and some UK Funder guidelines, but the absence of an expert panel discussion and published literature on the role of an independent PPI contributor was notable. Buck et al. reviewed planned PPI activities in NIHR HTA funded trials. When a PPI contributor was a member of the TSC, his/her role was unclear to all involved, including the PPI contributors themselves [23]. The preferred role of PPI contributors was in managerial or responsive roles, and these roles were also found to be more likely to achieve impact compared to involvement in oversight roles including the TSC [24]. In addition, a survey of UK CTUs demonstrated that identification of an appropriate PPI contributor was challenging [22]. The role, expected contributions, and need for independence of public contributors in the oversight of clinical trials warrant further investigation.

## Conclusion

Discussion among members of the expert panel and the study team has provided valuable information, based on real-life experience, and the recommendations made will contribute to the re-development and expansion of the MRC guidelines [1]. Some uncertainties could not be resolved in the expert panel meetings; one uncertainty, in particular, was the identification of examples of complex ethical issues and the training requirements of independent members. These, together with the role of public contributors as independent TSC members, merit further consideration. Experiences shared at the expert panel meetings were those of involvement in UK publically funded trials. Whilst many of the issues discussed are relevant beyond this scope, the transferability of recommendations will be dependent on commercial and international oversight requirements for randomised controlled trials.

## Additional file

Additional file 1:
**Summary of information provided to members of the expert panel.** (DOCX 35 kb)

## Abbreviations

CI: chief investigator
CTU: clinical trials unit
DMC: data monitoring committee
EME: efficacy and mechanism evaluation
HTA: health technology assessment
MRC: Medical Research Council
NIHR: National Institute of Health Research
PPI: patient and public involvement
TMG: trial management group
TSC: trial steering committee
