# Supplementary material for: Exploring the role and function of trial steering committees: results of an expert panel meeting
Source: Trials. 2015 Dec 30;16:597. doi: 10.1186/s13063-015-1125-z (PMC4696246; doi:10.1186/s13063-015-1125-z)
Supplement: Additional file 1: — Summary of information provided to members of the expert panel. (DOCX 35 kb) [file 13063_2015_1125_MOESM1_ESM.docx]

**Additional File 1. Summary of information provided to members of the expert panel.**

| **1** | **What is the role/remit of the TSC ?** |
| --- | --- |

***The guidelines say:***

| DAMOCLES | **On TSCs:**  Not discussed  **On DMCs to consider transferability:**  A diagram can help to clarify relationships when there are several inter-related committees. A short statement of the responsibilities of the other committees should be given if these are not provided in the protocol.  It is customary that the DMC does not make decisions about the trial, but rather makes recommendations to an appropriate executive committee or its Chair. |
| --- | --- |
| MRC Guidelines for GCP | Role  The role of the TSC is to provide overall supervision of the trial on behalf of the MRC.  It is the role of the TSC to monitor the progress of the trial and to maximise the chances of completing the study within the time scale agreed by the board.  Expectation   - To consider the recommendations of the Data Monitoring and Ethics Committee. - To inform the Council and relevant research boards on the progress of the trial - To advise Council on publicity and the presentation of all aspects of the trial |
| NIHR HTA Programme | Role  The role of the TSC is to provide overall supervision for a trial on behalf of the Trial Sponsor and Trial Funder and to ensure that the trial is conducted to the rigorous standards set out in the MRC Guidelines for GCP.  Expectations  One of the main features of the TSC is to provide advice, through its Chair, to the Chief Investigator(s), the Trial Sponsor, the Trial Funder, the Host Institution and the Contractor on all appropriate aspects of the trial. |
| HTA Research Governance Guidelines | *Same guidelines as NIHR HTA Programme above.* |
| EME Programme | *Same guidelines as NIHR HTA Programme above.* |

| **2** | **The makeup of the TSC** |
| --- | --- |

| a. | What should the membership of the TSC be with respect to size, members and the choice of Chair? |
| --- | --- |

***The guidelines say:***

| DAMOCLES | **On TSCs:**  Not discussed  **On DMCs to consider transferability:**  “..size is not likely to change the actual decision made but might affect the quality of the decision-making process”.  Choosing an odd number was seen as possibly helpful if voting was to be used in the decision making process. An appropriate range of membership was judged important, while keeping the size of the group manageable. Statistical and clinical inputs were deemed essential. Inclusion of more than one clinician could be appropriate; one of the case studies emphasised that over-dependence (or perceived over-dependence) on one clinician can unduly affect decision making. Although individuals with skills in key specialties are essential, the review of published work on small groups suggested that diversity is likely to improve decision making, provided that conflict is handled appropriately. No consensus was reached about ethicist or consumer or lay membership.  DMC members saw the appointment of the Chair of the DMC as crucial. They thought that the Chair should have previous experience of DMC meetings, an understanding of both clinical and statistical issues, experience of chairing meetings, and the ability to facilitate effective interaction in the group. Results from the small group processes review suggested that the Chair can have a big effect on decision outcome and the quality of decision making. Further, sound decision making was more likely if the Chair was facilitating (rather than directive) and impartial (being open to others’ opinions and not expressing their own views until after a full discussion). The planned membership and size of the DMC should, therefore, be outlined together with a description of how the Chair is to be chosen.  The Chair should have previous experience of serving on DMCs and experience of chairing and should be able to facilitate and summarise discussions. The Chair is sometimes chosen by the sponsor or the investigators running the trial and sometimes by the DMC members themselves. The Chair is expected to facilitate and summarise discussions.  It may be helpful to provide the trial coordinating centre with brief personal details (say, one paragraph) of all DMC members especially relating to experience relevant to the trial and to the operation of DMCs (such information need not be contained within the Charter). |
| --- | --- |
| MRC Guidelines for GCP | The membership should include an independent Chairman, not less than two other independent members and one or two Principal Investigators. Where possible membership should include a lay/consumer representative. |
| NIHR HTA Programme | The TSC will comprise:   - An independent chair (UK based and/or holding a substantive UK base appointment) - Independent clinicians with relevant expertise - Independent statisticians/epidemiologists/diagnosticians with relevant expertise - At least one individual who is able to contribute a patient and/or wider public perspective |
| HTA Research Governance Guidelines | Membership of the TSC should be limited and include an independent Chair, at least two other independent members, one or two principal investigators and, where possible, a consumer representative.  Involvement of independent members provides protection for both Trial Participants and Principal Investigators. |
| EME Programme | *Same guidelines as HTA Research Governance guidelines above.* |

| **2** | **The makeup of the TSC** |
| --- | --- |

| b. | Do TSCs always need independent members and, if so, how many? |
| --- | --- |

***The guidelines say:***

| DAMOCLES | **On TSCs:**  Not discussed  **On DMCs to consider transferability:**  Involvement of independent members should protect against biased decision making.  The members should be independent of the trial (eg, should not be involved with the trial in any other way or have some competing interest that could impact on the trial). Any competing interests, both real and potential, should be declared. A short competing interest form should be completed and returned by the DMC members to the trial coordinating centre (Annex 1). |
| --- | --- |
| MRC Guidelines for GCP | All TSCs are to have an independent Chair and at least 3 other independent members (see 3i). |
| NIHR HTA Programme | All TSCs are to have an independent Chair, independent clinicians and independent statisticians/epidemiologists/diagnosticians.  All TSCs are to have a minimum of a 75% majority of independent members. |
| HTA Research Governance Guidelines | All TSCs should include independent Chair and at least two other independent members. |
| EME Programme | *Same as HTA Research Governance guidelines above.* |

| **2** | **The makeup of the TSC** |
| --- | --- |

| c. | What is the definition of independence? |
| --- | --- |

***The guidelines say:***

| DAMOCLES | **On oversight committees:**  Define independent committees (or committee members) as committees (or committee members) completely uninvolved in the running of the trial and who cannot be unfairly influenced (either directly or indirectly) by people, or institutions, involved in the trial.  **On DMCs to consider transferability:**  Of 19 organisations with independent DMCs. Two referred to independence from the trial, and 11 specified that this meant independence from the clinical investigators and trial participants. Four specified independence from host institution, three from the TSC and one from the sponsor. There was some overlap in these responses. |
| --- | --- |
| MRC Guidelines for GCP | Defines independence as not involved directly in the trial other than as a member of the TSC. |
| NIHR HTA Programme | The definition of independence is as follows:   - Not part of the same institution as any of the applicants or members of the project team - Not part of the same institution that is acting as a recruitment or investigative centre - Not related to any of the applicants or members of the project team - For the chair only – not an applicant on a rival proposal |
| HTA Research Governance Guidelines | As for MRC Guidelines for GCP |
| EME Programme | As for MRC Guidelines for GCP |

| **2** | **The makeup of the TSC** |
| --- | --- |

| d. | Should funders be able to reject TSC members? |
| --- | --- |

***The guidelines say:***

| DAMOCLES | **On TSCs:**  Not discussed  **On DMCs to consider transferability:**  The Chair is sometimes chosen by the sponsor or the investigators running the trial and sometimes by the DMC members themselves. |
| --- | --- |
| MRC Guidelines for GCP | Applicants should submit a suggested membership with their full application.  The relevant Board(s) will decide on the final membership of the TSC. |
| NIHR HTA Programme | The NIHR HTA Programme Director will vet the nominees for TSC members and the TSC Chair submitted by the CI and formally appoint the chair and members by letter.  An indication of any proposed overseas members should be given at the full application stage and feedback on such proposals supplied following the Commissioning Board’s consideration for the application. |
| HTA Research Governance Guidelines | *Same guidelines as NIHR HTA Programme above.* |
| EME Programme | *Same guidelines as NIHR HTA Programme s above.* |

| e. | What, if any, are the implications of the same statistician being involved in production of DMC report (knowledge of unblinded comparative data), and being on the TSC? |
| --- | --- |

***The guidelines say:***

| DAMOCLES | **On TSCs:**  Some have suggested that the trial statistician remain blinded and that an independent unblinded statistician should be employed as the analysis statistician to carry out interim analysis and to work with the DMC. There is a concern that the trial statistician cannot participate neutrally in steering committee discussion, for example, relating to changing the primary outcomes, if seeing the accumulating data. A suggestion in the recent draft FDA guidelines to have an independent statistician has caused considerable consternation. For example, in a session of the Society for Clinical Trials annual meeting in 2002, concern was expressed that while the trial statistician knows the data set thoroughly, someone brought in from outside would find it difficult to produce the necessary analyses and may miss important information. |
| --- | --- |
| MRC Guidelines for GCP | Not discussed |
| NIHR HTA Programme | Not discussed |
| HTA Research Governance Guidelines | Not discussed |
| EME Programme | Not discussed |

| **3** | **What sort of experience, training or preparation should TSC members have?** |
| --- | --- |

***The guidelines say:***

| DAMOCLES | **On TSCs:**  Not discussed  **On DMCs to consider transferability:**  For DMCs the chair is likely to be most effective if he or she is experienced and understands both statistical and clinical issues. There was no evidence available to judge approaches to training.  It is generally accepted that experience of clinical trials and knowledge of the disease area of the trial are essential for DMC members, little has been written as to how individuals should gain suitable experience to serve as DMC members. Similarly, little has been published as to whether any formal training is required. |
| --- | --- |
| MRC Guidelines for GCP | Not discussed |
| NIHR HTA Programme | The clinicians, statisticians, epidemiologists, diagnosticians will have relevant expertise.  The Chair is required to provide an experienced opinion if conflicts arise. |
| HTA Research Governance Guidelines | Not discussed |
| EME Programme | Not discussed |

| **4** | **What is the role of the TSC prior to trial recruitment?** |
| --- | --- |

***The guidelines say:***

| DAMOCLES | **On TSCs:**  A DMC should be appointed before the trial begins. This allows them to meet in person, with representatives of the Steering Committee and the sponsor to learn about the trial and to review the protocol while there remains a chance to make changes. In reviewing the protocol, the DMC needs to be comfortable about the purpose, the methodology and ethical aspects of the trial, but should accept that responsibility for detail rests with the Steering Committee. |
| --- | --- |
| MRC Guidelines for GCP | At the first TSC meeting, targets for recruitment, data collection, compliance etc. should be agreed with the investigators.  The full protocol should be presented as an agenda item at the first TSC meeting to be agreed. |
| NIHR HTA Programme | The Chair of the TSC is responsible for arranging an inaugural meeting to finalise the protocol and set up a schedule of meetings to align with the project plan. |
| HTA Research Governance Guidelines | Not discussed |
| EME Programme | Not discussed |

| **5** | **Material available to a TSC (meeting report contents)** |
| --- | --- |

| a. | Should a TSC see any data split by treatment groups |
| --- | --- |

***The guidelines say?***

| DAMOCLES | **On TSCs:**  Open sessions: Accumulating information relating to recruitment and data quality (e.g. data return rates, treatment compliance) will be presented. Toxicity details based on pooled data will be presented and total numbers of events for the primary outcome measure and other outcome measures may be presented, at the discretion of the DMC.  There is a unanimity that the interim data and the deliberations of the DMC should be absolutely confidential. At the end of the meeting the DMC will make a recommendation to the steering committee, but the DMC will not discuss the actual data with the steering committee or anyone else. Breaches of confidence will be treated very seriously.  Pocock discussed the question of whether the pooled outcome data from the two arms of a trial might be released to trial investigators. “Such knowledge satisfies curiosity and instils confidence that a trial is functioning well, but it could adversely affect continuation? Knowledge that the total of primary events was well below twice that expected in the control group might lead to (possibly false) speculation that the treatment was effective, in which case such notes should not be released.” He notes, however, that a referee of his paper disagreed, “arguing that a steering committee cannot steer appropriately without access to such information”. |
| --- | --- |
| MRC Guidelines for GCP | The TSC should agree a set of data, based on the targets set, that should be presented to each TSC (template provided).  No one involved with the TSC should be present at the DMC meeting to see unblinded data. |
| NIHR HTA Programme | Not discussed |
| HTA Research Governance Guidelines | Not discussed |
| EME Programme | Not discussed |

| **5** | **Material available to a TSC (meeting report contents)** |
| --- | --- |

| b. | Should external evidence be incorporated and how? |
| --- | --- |

***The guidelines say:***

| DAMOCLES | **On TSCs:**  Not discussed  **On DMCs to consider transferability:**  Identification and circulation of external evidence (e.g. from other trials/systematic reviews) is not the responsibility of the DMC members. The PI or the trials office team will usually collate (e.g. from other trials) any such information. |
| --- | --- |
| MRC Guidelines for GCP | The TSC should consider new information relevant to the trial including question s from the DMC and the results from other studies. It is the responsibility of the PI and Chairman and other independent members of the TSC to bring to the attention of the TSC any results from other studies that may have a direct bearing on future conduct of the trial. |
| NIHR HTA Programme | It is the role of the TSC to consider new information or relevance to the research question. |
| HTA Research Governance Guidelines | *Same guidelines as NIHR HTA Programme above.* |
| EME Programme | *Same guidelines as NIHR HTA Programme above.* |

| c. | How does this material differ for TSCs that cover multiple trials? |
| --- | --- |

***The guidelines say:***

| DAMOCLES | **On TSCs:**  Not discussed  **On DMCs to consider transferability:**  Interaction between DMCs that are monitoring similar trials is generally regarded as useful, in principle. However, the practicalities of such interaction, including timing of reports, meetings and relevant information is difficult. Perhaps more importantly, this sharing erodes the independence of each trial, and thus prevents ‘independent confirmation’ of results; such independence is an important, basic tenet of the scientific method. Other difficulties are that interaction between the DMC, those preparing the report and the trial investigators is important in making any major decisions and this would be difficult or impossible in a model where DMCs are sharing confidential data. In the absence of such interaction it is entirely possible that one DMC may misinterpret or overinterpret a written report. A closely allied difficulty is that it is not clear what should happen if the DMCs disagree, especially if the DMCs are representing different communities (e.g. the trials are in different countries). It is likely that it would be difficult to include the trial investigators in any decisions made. As a consequence, sharing of data is rarely done in practice. For similar reasons, it is generally not recommended that the same individual serves on two parallel DMCs of two independent similar trials. |
| --- | --- |
| MRC Guidelines for GCP | Not discussed |
| NIHR HTA Programme | Not discussed |
| HTA Research Governance Guidelines | Not discussed |
| EME Programme | Not discussed |

| **6** | **What are the questions that the TSC should not ask the DMC to make recommendations upon, for example change to primary outcome or target sample size (other than pre-planned internal pilot)?** |
| --- | --- |

***The guidelines say:***

| DAMOCLES | | **On TSCs:**  Not discussed |
| --- | --- | --- |
| MRC Guidelines for GCP | | The DMC is required to consider any requests for release of interim trial data and to recommend to the TSC on the advisability of this.  In the event of further funding being required, the DMC should provide the TSC with appropriate information and advice on the data gathered to date that will not jeopardise the integrity of the study. |
| NIHR HTA Programme | | The DMC may be asked by the TSC to consider data emerging from other related studies.  If funding is required above the level originally requested, the DMC may be asked by the TSC to provide advice and, were appropriate, information on the data gathered in a way that will not compromise the trial. |
| HTA Research Governance Guidelines | | Not discussed |
| EME Programme | | Not discussed |
|  | |  |
| **7** | **If there is no DMC what is the impact on the role/remit of the TSC?** | |

***The guidelines say:***

| DAMOCLES | **On TSCs:**  Not discussed  **On DMCs to consider transferability:**  Reasons are provided in the report for not having a DMC. The reason provided: the TSC discussed the issue and decided that there was no need for a DMC, for example owing to the nature of the intervention, which might be minor or involve minimal risk to patients. |
| --- | --- |
| MRC Guidelines for GCP | Not discussed |
| NIHR HTA Programme | Not discussed |
| HTA Research Governance Guidelines | Not discussed |
| EME Programme | Not discussed |

| **8** | **How should decisions be reached within the TSC?** |
| --- | --- |

***The guidelines say:***

| DAMOCLES | **On TSCs:**  Not discussed  **On DMCs to consider transferability:**  It has been suggested that, ideally all-important DMC decisions should be reached by consensus rather than by a majority vote. However, on occasions, arriving at a resolution of a difficult issue may require a vote, and so it is recommended by some that the DMC should have an odd number of members. Some DMCs allow the chair a casting vote to cover the eventuality of equal votes. It has been recommended that the process of decision-making should be laid out in advance (e.g. DMC charter), including, for example, when a DMC is quorate for decision-making. |
| --- | --- |
| MRC Guidelines for GCP | Not discussed |
| NIHR HTA Programme | Only appointed members will be entitled to vote and the chair will have casting vote.  The minimum quoracy for a meeting to conduct business is 67% of appointed members. |
| HTA Research Governance Guidelines | Not discussed |
| EME Programme | Not discussed |

| **9** | **How should ethical issues be handled in TSCs? For example if higher rates of adverse events were observed in one site/surgeon or an occurrence of a potential serious breach e.g. dosing** |
| --- | --- |

***The guidelines say:***

| DAMOCLES | **On TSCs:**  Not discussed |
| --- | --- |
| MRC Guidelines for GCP | The PI is required to submit an annual report to the MRC based on template provided. The report should be endorsed by the TSC and inform of any new information that has a bearing on safety or ethical acceptability. |
| NIHR HTA Programme | A main feature of the TSC is to ensure appropriate ethical and other approvals are obtained in line with the project plan |
| HTA Research Governance Guidelines | Not discussed |
| EME Programme | Not discussed |

| **10** | **What should be done in difficult or complex situations?** |
| --- | --- |

***The guidelines say:***

| DAMOCLES | **On oversight committees:**  If the DMC is advisory (rather than executive) then to ensure that conflicts of interest do not dominate, it has been suggested that the DMC should report to the sponsor and trial investigators (in the form of a representative committee, such as an executive or a steering committee) simultaneously. In the lead up to a decision there would be a discussion including both the investigators and the sponsor, or their representatives. One model is that it is the sponsor who takes the final decision, because the legal obligation for safety of the participants falls to them. Another is that it is a joint decision through a formal committee containing both the sponsor and investigators which is formed to receive such reports. |
| --- | --- |
| MRC Guidelines for GCP | Not discussed |
| NIHR HTA Programme | The Chair is responsible for providing an independent, experienced opinion if conflicts arise between the needs of the research team, the funder the sponsor, the participating organisations and/or any other agencies. |
| HTA Research Governance Guidelines | Not discussed |
| EME Programme | Not discussed |

| a. | When there is a recommendation from the DMC to stop the trial, who should be unblinded and what are the implications if the trial then doesn’t stop? |
| --- | --- |

***The guidelines say:***

| DAMOCLES | **On oversight committees:**  The DMC may allow “selected individuals” (e.g. the chair of the steering committee) to become unblinded with respect to certain specific results if, in the view of the DMC, the trial – and particular patient safety – would be better served by such action.  The DMC usually makes recommendations to the trial’s steering committee. In practice, a recommendation to cease recruitment is effected by unblinding the steering committee to the latest efficacy and safety data on which such a recommendation was based. In most cases the steering committee will follow the recommendation of the DMC, but on occasion they will not (examples given in paper). Using this approach, it is for the steering committee, not the DMC, to decide to whom the trial results should be released, although the DMC may have a say.  If the trial steering committee (TSC) or sponsor disagrees with the DMC the “In the absence of ethical issues the sponsor might freely override a DMC decision for early termination”. Where the investigators do not choose to follow the course of action suggested by the DMC it can lead to a great deal of friction, and sometimes the resignation of DMC members. It has been suggested that “a DMC charter should contain an ‘escalation clause’ for resolving differences of opinion”. If a TSC, sponsor or coordinators were seriously concerned about a recommendation of the DMC they could instead “convene another committee” |
| --- | --- |
| MRC Guidelines for GCP | Not discussed |
| NIHR HTA Programme | The Chair is responsible to comment in detail regarding the continuation or termination of the project. |
| HTA Research Governance Guidelines | Not discussed |
| EME Programme | Not discussed |

| **10** | **What should be done in difficult or complex situations?** |
| --- | --- |

| b. | Decisions on site closures guided by performance |
| --- | --- |

***The guidelines say:***

| DAMOCLES | **On TSCs:**  Not discussed |
| --- | --- |
| MRC Guidelines for GCP | Not discussed |
| NIHR HTA Programme | Not discussed |
| HTA Research Governance Guidelines | Not discussed |
| EME Programme | Not discussed |

| c. | When funders withdraw/stop funding? |
| --- | --- |

***The guidelines say:***

| DAMOCLES | **On TSCs:**  Not discussed |
| --- | --- |
| MRC Guidelines for GCP | If progress on the trial suggests that an extension may be necessary, the TSC should notify MRC officers. |
| NIHR HTA Programme | The Chair is responsible to comment on any extension requests and, where appropriate, provide a letter of recommendation to accompany such a request. |
| HTA Research Governance Guidelines | The TSC will provide evidence to support any requests for extensions, indicating that all practical steps have been taken to achieve targets. |
| EME Programme | The TSC will be asked to comment in detail on extension requests. |

| d. | When an external body wants access to the data while the trial is on-going? For example (i) to design a new trial or (ii) there is interest from industry |
| --- | --- |

***The guidelines say:***

| DAMOCLES | **On TSCs:**  Not discussed  **On DMCs to consider transferability:**  There was considerable variation in the investigators’ opinion of whether a DMC should share information with other bodies, for example with the DMC of a similar trial. 2 (of 20) investigators suggested that any request for the DMC to share information should be passed on to the TSC, which would make a decision based on the individual case.  The DMC is required to consider any requests for release of interim trial data and to recommend to the TSC on the advisability of this.  In the event of further funding being required, the DMC should provide the TSC with appropriate information and advice on the data gathered to date that will not jeopardise the integrity of the study. |
| --- | --- |
| MRC Guidelines for GCP | Not discussed |
| NIHR HTA Programme | Not discussed |
| HTA Research Governance Guidelines | Not discussed |
| EME Programme | Not discussed |

| **11** | **What should the TSCs role be concerning publications? Please consider publications prior to the main trial report and subsequent articles.** |
| --- | --- |

***The guidelines say:***

| DAMOCLES | **On TSCs:**  The responsibility for publishing findings is considered to belong to the study sponsor, the study’s Executive Committee or publication committee, and the principal investigators.  In a UK MRC model, responsibility for publishing trial results when the trial mechanism fails should be assumed by the steering committee.  **On DMCs to consider transferability:**  At the end of the trial there may be a meeting to allow the DMC to discuss the final data with principal trial investigators/sponsors and give advice about data interpretation The DMC may wish to see a statement that the trial results will be published in a correct and timely manner.  The information about the DMC members should be named and their affiliations listed in the main report, unless they DMC that will be included in explicitly request otherwise. A brief summary of the timings and conclusions of DMC meetings published trial reports should be included in the body of this paper.  Whether the DMC will have the The DMC may wish to be given the opportunity to read and comment on any publications opportunity to approve before submission. |
| --- | --- |
| MRC Guidelines for GCP | Not discussed |
| NIHR HTA Programme | Not discussed |
| HTA Research Governance Guidelines | Not discussed |
| EME Programme | Not discussed |

| **12** | **What should the TSCs role be concerning requests for data sharing? Please consider requests prior to and following the main publication?** |
| --- | --- |

***The guidelines say:***

| DAMOCLES | **On TSCs:**  Not discussed |
| --- | --- |
| MRC Guidelines for GCP | Not discussed |
| NIHR HTA Programme | Not discussed |
| HTA Research Governance Guidelines | Not discussed |
| EME Programme | Not discussed |
